# Influence of the calcium concentration in the presence of organic phosphorus on the physicochemical compatibility and stability of all-in-one admixtures for neonatal use

**DOI:** 10.1186/1475-2891-8-51

**Published:** 2009-10-26

**Authors:** Daniela de Oliveira Ribeiro, Bianca Waruar Lobo, Nádia Maria Volpato, Venício Féo da Veiga, Lúcio Mendes Cabral, Valeria Pereira de Sousa

**Affiliations:** 1 Departamento de Medicamentos, Faculdade de Farmácia, Centro de Ciências da Saúde, Universidade Federal do Rio de Janeiro, CCS, Bloco B ss sala 15, Rio de Janeiro, RJ 21941-902, Brazil; 2 Instituto de Microbiologia Professor Paulo de Góes, Centro de Ciências da Saúde, Universidade Federal do Rio de Janeiro, Rio de Janeiro, RJ 21941-902, Brazil

## Abstract

**Background:**

Preterm infants need high amounts of calcium and phosphorus for bone mineralization, which is difficult to obtain with parenteral feeding due to the low solubility of these salts. The objective of this study was to evaluate the physicochemical compatibility of high concentrations of calcium associated with organic phosphate and its influence on the stability of AIO admixtures for neonatal use.

**Methods:**

Three TPN admixture formulas were prepared in multilayered bags. The calcium content of the admixtures was adjusted to 0, 46.5 or 93 mg/100 ml in the presence of a fixed organic phosphate concentration as well as lipids, amino acids, inorganic salts, glucose, vitamins and oligoelements at pH 5.5. Each admixture was stored at 4°C, 25°C or 37°C and evaluated over a period of 7 days. The physicochemical stability parameters evaluated were visual aspect, pH, sterility, osmolality, peroxide formation, precipitation, and the size of lipid globules.

**Results:**

Color alterations occurred from the first day on, and reversible lipid film formation from the third day of study for the admixtures stored at 25°C and 37°C. According to the parameters evaluated, the admixtures were stable at 4°C; and none of them presented precipitated particles due to calcium/phosphate incompatibility or lipid globules larger than 5 μm, which is the main parameter currently used to evaluate lipid emulsion stability. The admixtures maintained low peroxide levels and osmolarity was appropriate for parenteral administration.

**Conclusion:**

The total calcium and calcium/phosphorus ratios studied appeared not to influence the physicochemical compatibility and stability of AIO admixtures.

## Background

Preterm infants, children born before 37 weeks of pregnancy, are the major recipients of parenteral nutrition therapy. This group is frequently intolerant to enteral feeding due to anatomic and functional immaturity of the digestive tract, added to other clinical conditions that affect cardiovascular function in the preterm post-natal life [1-3]. These patients need a calcium/phosphorus ratio higher than 1 (42 mg/Kg/d Ca and 36 mg/Kg/d P) in order to allow bone mineralization. Studies on calcium and phosphorus retention in neonates propose that 1.7/1 (75 mg/Kg/d Ca and 45 mg/Kg/d P) calcium/phosphorus ratio is closer to that observed for intra-uterine life, allowing greater retention of these ions [4]. This ratio is difficult to obtain with parenteral feeding, because the availability of these ions offer is limited by their salts solubility in the formulation [5].

The most daunting problem today for pharmaceutical preparation practices of parenteral nutrition is related to the day-to-day change of volume and formulation nutrients, due to changing clinical conditions and maturation. Considering the low volumes used in neonatology, it is a concern to ensure both physicochemical compatibility and adequate calcium and phosphorus supply. The compatibility depends on factors such as solution pH, temperature, material of the parenteral nutrition container, oxygen, light exposure, composition of trace elements, presence of vitamins, peroxidation, relative concentration of each ion, and order of addition of divalent ions such as calcium [6,7].

Elevated concentrations of cationic electrolytes such as calcium also interfere in the total parenteral nutrition (TPN) admixture stability and may influence directly the size of lipid globules, contributing to the formation of unstable phases in the lipid emulsion (LE) such as aggregation, flotation, coalescence and phase separation [8]. The main parameter currently used to evaluate lipid emulsion physicochemical stability is the percentage of particles larger than 5 μm. According to pharmacopeia specifications, this should not exceed a total of 0.05% of lipid globules in the admixture [9,10]. Lipid globules of this size may clog the pulmonary capillaries, producing an embolic syndrome and causing cell death mainly in preterm infants [11,12]. In addition, an incompatibility between calcium and phosphorus parenteral salts, forming an insoluble dibasic calcium phosphate precipitate, may similarly produce a potentially fatal respiratory arrest [13].

Organic phosphate was introduced on the pharmaceutical market about two decades ago, and was immediately approved in some European countries. Due to the high cost, its use was restricted to patients such as preterm infants who needed high calcium and phosphorus concentrations [14]. The use of organic phosphate in TPN is not permitted in the United States [15], so compatibility and stability studies on the TPN admixture in this country are exclusively associated with preparations containing inorganic phosphate [16].

Since the introduction of organic phosphate on the market, some authors have shown, in clinical comparative studies, the benefits of the use of organic phosphate in relation to inorganic phosphate [4,17,18]. However, there are divergences concerning the utilization of this product, mainly in regard to the physicochemical TPN admixture compatibility with high calcium levels, as well as bioavailability. In addition there is still the possible interference of high calcium concentrations, which may also affect the emulsion stability.

Due to the clinical relevance of compatibility with high calcium and organic phosphate concentrations and emulsion stability issues posed by the action of calcium and other cationic electrolytes upon negatively charged lipid droplets, this paper was aimed at evaluating these factors in neonatal all-in-one admixtures. The goal was to attain a higher safety level for these preparations and safe concentrations in TPN admixtures.

## Methods

### Preparation of the admixtures studied

Three TPN admixture formulas for neonatal use were prepared aseptically in a 300-ml 3-layered bag composed by polyester, polypropylene and polyethylene (HalexIstar, Goiânia, Brazil) under a laminar-flow hood in accordance with the National Health Department specifications Nr. 272 (1988), designed for infusion through central access. The TPN admixture formulas were prepared with market products from pharmaceutical industries and based on official regulations [13,16,19].

### Experimental Procedures

Each admixture was prepared separately in triplicate or quadruplicate (referred to as 3 or 4 lots) divided into three groups and stored at different temperatures: 4°C ± 2°C, in refrigerator; 25°C ± 3°C, simulating room temperature; and in an incubator at 37°C ± 2°C, simulating an infusion temperature in a worst-case scenario. A different number of samples were tested from each of the lots depending on the technique applied. The experiments were performed on the day of the TPN admixture preparation and also 24 h, 48 h, 72 h and 7 days after preparation, time periods indicated as D0, D1, D2, D3 and D7, respectively.

Samples for the sterility test and for the physicochemical tests were aseptically collected from each formulation at appropriate intervals using a plastic syringe. The physicochemical compatibility and stability parameters evaluated were general visual aspect, presence of dibasic calcium phosphate precipitation, pH, sterility, osmolarity, peroxide formation, and the size of lipid globules.

### Sterility test

The maintenance of the TPN admixture sterility was evaluated using a biphasic culture medium, which contains a liquid phase composed of sterile soybean casein broth and a solid phase composed of sterile soybean casein agar (TSA/TSB). This culture medium promotes growth of non-specific aerobic microorganisms and is used in accordance with recommendations of USP 31, chapter 71 [19,20]. The analysis is based on inoculation of a TPN admixture sample (2 ml) in a flask containing the biphasic culture medium at the end of the manipulation of each bag. This flask was immediately stored after inoculation in an oven at 30°C ± 2°C for a 14-day period. At intervals (24 h) during the incubation period, the media were examined for macroscopic evidence of microbial growth in the solid phase. If no evidence of microbial growth was found after 14 days the product complies with the test for sterility. This procedure was carried out in all admixture lots.

### Physicochemical assessments

On visual inspection, parameters that evidence instability and incompatibility events were examined. Color change, precipitation, film formation and phase separation were sought. The color change assay was not graduated; instead, a positive response was registered when darkening occurred. Precipitation was evaluated visually and microscopically by observing formation of rigid crystalline structures. The stability of the emulsion, on a visual basis, was assessed by the observation of a yellow fat layer on the top of the admixture, referred to as film formation (creaming). The film thickness was measured using a ruler with millimeter precision. Phase separation was recorded when two different phases could be observed. The visual inspection was performed in all the admixtures during the seven days of study at all temperatures and was registered through digital photography.

For the evaluation of pH, a Mettler Toledo potentiometer calibrated with pH 4 and pH 7 buffers was used. For each measurement, a 10 ml sample was collected and placed in an amber glass flask. The pH was measured by dipping the electrode directly into the emulsion, at room temperature. The visual inspection and pH determination were carried out each day in quadruplicate for each formulation in all the conditions studied.

The osmolalities were measured experimentally using a Wescor micro-osmometer. The TPN admixture osmolalities is determined through its freezing point. Before each measurement session, the equipment was calibrated with standard solutions of 100, 290 and 1000 mOsm/Kg H_2_O, composed of NaCl and preservative in water. The equipment range is from 0 to 3000 mOsm/Kg H_2_O. The osmolality (mOsm/Kg H_2_O) was converted into osmolarity (mOsm/l H_2_O) using the density of the TPN admixtures measured with a pycnometer. The mean density of the admixtures studied was 1.04 g/ml at 25°C. The osmolarity of the TPN admixtures was estimated by multiplying the measured osmolality by the TPN admixture density and subtracting the concentration (g/ml) of the solutes present in the emulsion, according to Martin *et al *[20]. The concentration of the solutes calculated for the TPN admixture was 0.2 g/ml at the highest concentration of calcium. The measurements were carried out on three different lots on day 0 for each formulation at each temperature condition.

### Globule size measurement

The size of the lipid globules was measured with an Axioplan 2 optical microscope (Carl Zeiss, Germany) using a digital camera connected to a computer and a television set, at a total magnification of 1000×. This combination makes it possible to measure globules as small as 0.2 μm. A 5-ml sample of TPN admixture was collected from the bag with a sterile syringe and placed in a sterile vacutainer. From this vacutainer, a 20-μl sample was collected and diluted 1:4 using its own admixture but without lipids. From this dilution, a 6-μl sample was transferred to a slide with an automatic pipette and then an 18 × 18 mm cover slip was laid over it. Initially different fields were examined to investigate incompatibility based on the presence of dibasic calcium phosphate crystals in the sample. When crystals were not found, evaluation of the lipid globule stability was initiated. Between 1000 and 1500 lipid globules per field were observed and counted in each of two fields in each admixture sample. The measurement was carried out with two different lots of each formulation for each of the studied conditions. For each lot, images of three randomly chosen fields were registered for posterior analysis by the software. Results are presented in the figure as a representative field from a set of three collected from each of two different lots, on different days. The globules are grouped according to size range: 0-1 μm; 1-2 μm; 2-3 μm and > 3 μm, and the percentage in each range are calculated in relation to the total number of measured globules. Maximum globule diameter was also recorded.

The images were collected and kept for further analysis. The images were treated with the *AnalySIS *software (Soft Imaging System, Münster, Germany), which converts the images into gray scales and then into a black and white binary system, allowing the identification and counting of the lipid globules.

### Peroxide formation

Lipid hydroperoxide and hydrogen peroxide were quantified by evaluating the oxidation of iron in a xylenol orange solution (Spectrum, New York, USA), modified for use with lipids according to the FOX 2 method previously described [21,22]. The method is based on the reaction of 2-thiobarbituric acid (TBA) with aldehydes formed by oxidized lipids in acid medium. The reaction is processed after 30 min, followed by centrifugation. This reaction forms a red-colored product, which is read at a wavelength of 560 nm in a Shimadzu model UV 2401PC spectrophotometer. It was tested all the admixtures through this methodology over a period of seven days at the temperatures of 4°C and 37°C totally photo protected (dark room), 25°C exposed to indirect artificial light, including the admixtures contained in bags photo protected at 25°C.

The peroxide concentration in the admixture samples was calculated from a hydrogen peroxide standard curve, in the range of 1 a 100 μM, prepared at the same day [21,23,24]. The experiments were carried out with 3 different lots (i.e. in triplicate).

### Statistical treatment

The results are expressed as mean and standard deviation. The graphical displays were created on SigmaPlot 10.0 (SPSS, Erkrath, Germany). The experimental results were analyzed for significance using Student's *t *test at P < 0.05.

## Results

Table 1 presents the three formulas, PC, P1 and P2, designed for this study. The TPN formulas differ in calcium content. The calcium/phosphorus ratios for P1 and P2 admixtures are (calcium and phosphorus) 2/1 and 4/1, respectively, and the control parenteral nutrition (PC) is free of calcium. Thus the calcium content of the admixtures was different in each formula, while the organic phosphate content was the same in the concomitant presence of vitamins and trace elements. All the nutrients were mixed in the same bag, always following the order presented at Table 1. The compatibility and stability of the admixtures were assessed.

**Table 1:** Formulations used in the study

**Insumes**	**Daily allowance**	**PC**	**P1**	**P2**
Total volume	100-150 mL/kg/d	100 ml	100 ml	100 ml
Pediatric amino acid with taurine 10%	1-3 g/kg/d	3 g	3 g	3 g
Multivitamin	A- 1.38 mg/d	0.128 mg	0.128 mg	0.128 mg
	D- 6.25 μg/d	0.5 μg	0.5 μg	0.5 μg
	E- 1.67 mg/d	1.0 mg	1.0 mg	1.0 mg
	B_1_- 11.25 mg/d	0.3 mg	0.3 mg	0.3 mg
	B_2_- 2.5 mg/d	0.36 mg	0.36 mg	0.36 mg
	B_3_- 25 mg/d	4.0 mg	4.0 mg	4.0 mg
	B_5_- 6.5 mg/d	1.5 mg	1.5 mg	1.5 mg
	B_6_- 3.0 mg/d	0.4 mg	0.4 mg	0.4 mg
	B_7_- --	6.0 μg	6.0 μg	6.0 μg
	B_9_- 200 μg/d	40 μg	40 μg	40 μg
	B_12_- 3.0 μg/d	0.5 μg	0.5 μg	0.5 μg
	C- 250 mg/d	10 mg	10 mg	10 mg
Glucose 50%	4-12 mg/kg/min	8.64 g	8.64 mg	8.64 mg
Pediatric element + zinc acetate	Zn- 400 μg/kg/d	350 μg	350 μg	350 μg
	Cu- 20 μg/kg/d	20 μg	20 μg	20 μg
	Cr- 0.2 μg/kg/d	0.2 μg	0.2 μg	0.2 μg
	Mn-1.0 μg/kg/d	2.0 μg	2.0 μg	2.0 μg
NaCl* 20%	3-5 mEq/kg/d	4 mEq	4 mEq	4 mEq
Sodium glycerophosphate	1-2 mEq/kg/d	1.1 mEq	1.1 mEq	1.1 mEq
KCl* 10%	2-3 mEq/kg/d	2 mEq	2 mEq	2 mEq
Calcium gluconate	200-800 mg/kg/d	0	500 mg	1000 mg
Calcium	18.6-37.2 mg/kg/d Ca	0	46.5 mg	93.0 mg
Magnesium sulphate	0.25-0.5 mEq/kg/d	0.25 mEq	0.25 mEq	0.25 mEq
Lipid emulsion 20% MCT/LCT*	1-3 g/kg/d	2 g	2 g	2 g

MCT/LCT- medium- and long-chain triglycerides; NaCl - sodium chloride; KCl -potassium chloride; Zn-Zinc; Cu-Copper; Cr-Chromium; Mn-Manganese; A-Retinol; D-Cholecalciferol; E-Tocopherol; B_1_-Thiamine; B_2_-Riboflavin; B_3_-Nicotinamide; B_5_-Pantothenic acid; B_6_-Pyridoxine; B_7_-Biotin; B_9_-Folic acid; B_12_-Cyanocobalamin; C-Ascorbic acid.

The following parameters were evaluated through visual inspection: color alterations, precipitation, film formation, and phase separation, for the three admixtures studied over a period of seven days at temperatures of 25°C, 4°C and 37°C, as demonstrated in Table 2. After 24 h at temperatures of 25°C and 37°C, color alteration was observed in all the formulations. On the other hand, there was no color alteration at any time for admixtures at 4°C. No precipitation due to incompatibility was observed in any of the admixtures. The formation of a film layer, the first step in a phase separation process, was observed only after 72 h and only for admixtures maintained at 25°C and 37°C. It is worth emphasizing that formation of this film was easily reversed with a smooth shake. No phase separation was observed in any of the admixtures.

**Table 2:** Visual inspection of TPN admixtures

**Parameters**	**PC**					**P1**					**P2**				
	
	**25°C**														
	
	D0*	**D1**	**D2**	**D3**	**D7**	**D0**	**D1**	**D2**	**D3**	**D7**	**D0**	**D1**	**D2**	**D3**	**D7**
Color alteration	A	**P**	**P**	**P**	**P**	A	**P**	**P**	**P**	**P**	A	**P**	**P**	**P**	**P**
Precipitation	A	A	A	A	A	A	A	A	A	A	A	A	A	A	A
Film formation	A	A	A	**P**	**P**	A	A	**P**	**P**	**P**	A	A	A	**P**	**P**
Phase separation	A	A	A	A	A	A	A	A	A	A	A	A	A	A	A
	
	**4°C**														
	
	**D0**	**D1**	**D2**	**D3**	**D7**	**D0**	**D1**	**D2**	**D3**	**D7**	**D0**	**D1**	**D2**	**D3**	**D7**

Color alteration	A	A	A	A	A	A	A	A	A	A	A	A	A	A	A
Precipitation	A	A	A	A	A	A	A	A	A	A	A	A	A	A	A
Film formation	A	A	A	A	A	A	A	A	A	A	A	A	A	A	A
Phase separation	A	A	A	A	A	A	A	A	A	A	A	A	A	A	A
	
	**37°C**														
	
	**D0**	**D1**	**D2**	**D3**	**D7**	**D0**	**D1**	**D2**	**D3**	**D7**	**D0**	**D1**	**D2**	**D3**	**D7**

Color alteration	A	**P**	**P**	**P**	**P**	A	**P**	**P**	**P**	**P**	A	**P**	**P**	**P**	**P**
Precipitation	A	A	A	A	A	A	A	A	A	A	A	A	A	A	A
Film formation	A	A	A	**P**	**P**	A	A	A	**P**	**P**	A	A	A	**P**	**P**
Phase separation	A	A	A	A	A	A	A	A	A	A	A	A	A	A	A

*D: day, A: absence, P: presence; n = 4 lots.

There were no colonies forming units (CFUs) on the solid phase in the culture medium after fourteen days of incubation in an oven at 30°C. The absence of CFUs in the biphasic culture medium demonstrates the maintenance of the admixtures' sterility.

The mean and standard deviation of pH values for TPN admixtures during the seven days of study, at the three different temperatures selected. In all cases the pH remained around 5.5 throughout the study period and showed no significant differences between the values of D7 and D0 in each condition tested. P2 admixture presented a initial pH of 5.57 ± 0,32; after seven days of storage at 25°C, 4°C and 37°C the values measured were: 5.53 ± 0.28, 5.51 ± 0.36 and 5.52 ± 0.30, respectively. Similar values were measured for PC and P1.

The osmolarity values for TPN admixtures are presented in Table 3. The results are presented separately according to temperature, since each TPN admixture corresponds to a different lot. The mean values showed no significant differences with temperature, also demonstrating homogeneity among the lots.

**Table 3:** Values of the experimental osmolarity for TPN admixtures at the three temperatures selected

	**Experimental Osmolarity (mOsm/L)**		
	**25°C**	**4°C**	**37°C**
	
**PC**	848 ± 8.7*	791 ± 2.4	792 ± 8.0
**P1**	791 ± 7.0	772 ± 1.8	805 ± 17
**P2**	831 ± 13	865 ± 14	836 ± 54

Mean ± standard deviation, n = 3 lots, measured on D0.

The presence of particles in the admixtures was evaluated by optical microscopy (OM). Particles are easily recognized due to the rigid crystalline structure of the precipitates. No evidence of precipitation was observed in the admixtures under any of the conditions studied.

**Figure 1:**
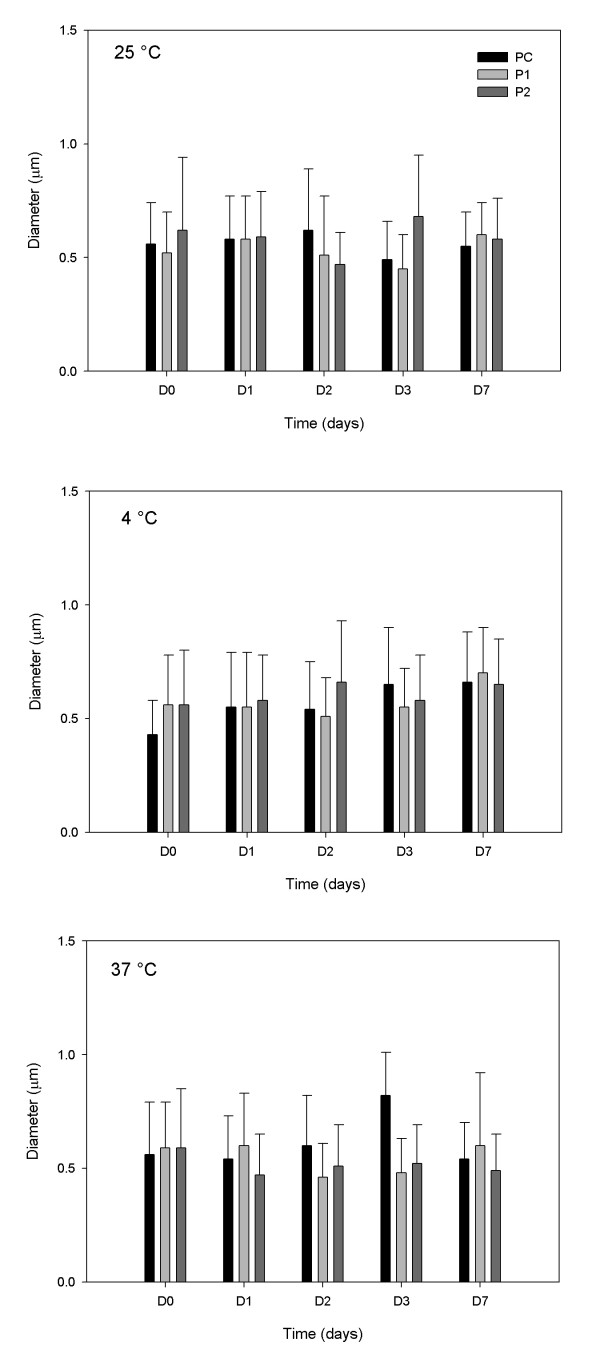
**Number of globules counted for each bar, use of OM**. Bar graphs represent the mean and standard deviation of the diameter of lipid globules present in admixtures PC, P1 and P2 stored at 25°C, 4°C and 37°C, during seven days of study D0 - D7 (n = 2 lots).

Among parameters obtained through OM, the one selected to evaluate the admixtures' stability was the diameter of the lipid globules. This parameter provides an index of the size of the lipid globules, making it possible to monitor size changes due to temperature and type of admixture over time. Figure 1 represents the mean diameter of the lipid globules over the seven days; maximum values are shown in Table 4, demonstrating that no globules greater than 4 μm were formed.

**Table 4:** Maximum lipid globule size diameter

**Temperature (°C)**	**Maximum diameter (μm)**				
	
	**D0**	**D1**	**D2**	**D3**	**D7**
	**PC**				
**25**	2.2	2.7	3.5	2.2	1.3
**4**	2.6	2.1	1.9	2.1	2.4
**37**	1.9	2.4	2.7	2.9	2.6
	
	**P1**				
	
**25**	2.3	1.9	2.3	1.6	3.8
**4**	2.4	2.9	2	1.5	1.8
**37**	2.1	1.6	2.2	1.8	3.1
	
	**P2**				
	
**25**	3.7	3	1.6	2.3	1.8
**4**	3.1	2.4	2.8	1.9	2
**37**	4	2.8	2.6	2.5	2.2

n = 2 lots for each condition at each time.

D0, D1, D2, D3 and D7 represents the day of the TPN admixture preparation and 24 h, 48 h, 72 h and 7 days after preparation, respectively.

Table 4 serves to emphasize that occasionally a larger lipid globule could be found, regardless of the day of analysis, as for example in D0, where there is a 4 μm lipid globule in admixture P2.

**Figure 2:**
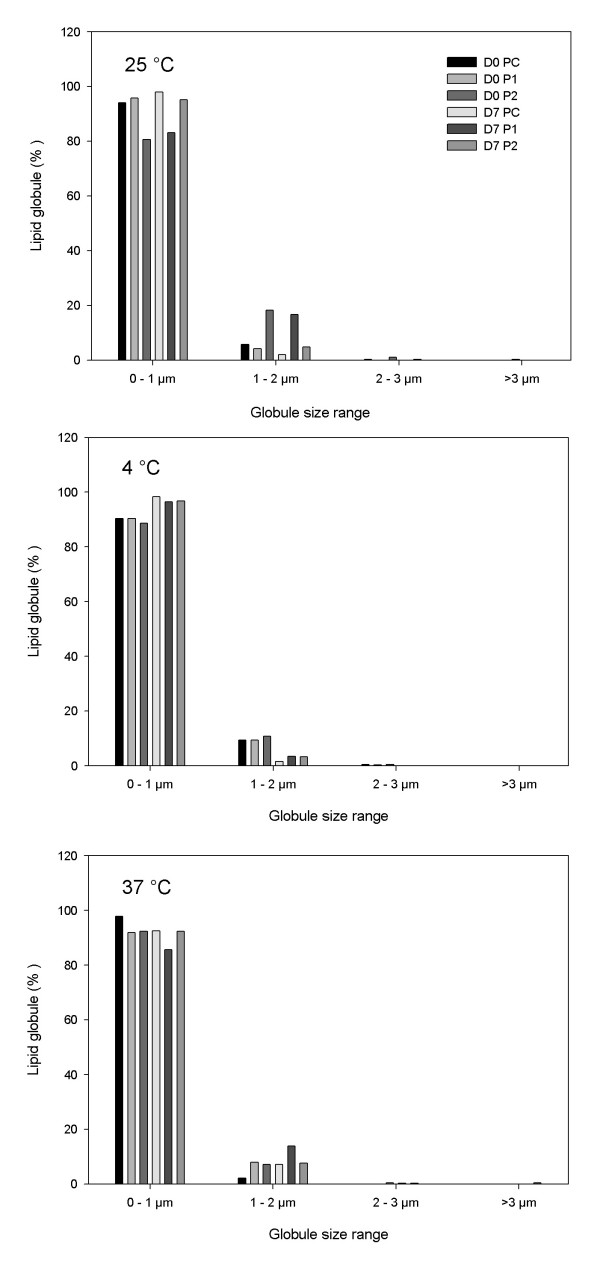
**Percentage of lipid globules in relation to total number of globules examined, distributed by size range**. Bar graphs represent the mean percentage of lipid globules in each range present in admixtures PC, P1 and P2 stored at 25°C, 4°C and 37°C after 0 and seven days of study (n = 2 lots).

Figure 2 shows the distribution of globule size range in percent of total number of globules measured under each condition on days 0 and 7. None of the lipid globules exceeded 4 μm in any of the admixtures during the seven days of analysis at any temperature, even in the presence of high amounts of calcium.

Other optical microscopy parameters related to stability such as Ferret diameter, area, sphericity, perimeter, convex perimeter, format factor and stretching were measured. There was no significant alteration in any of these parameters for any of the admixtures with time or temperature.

**Figure 3:**
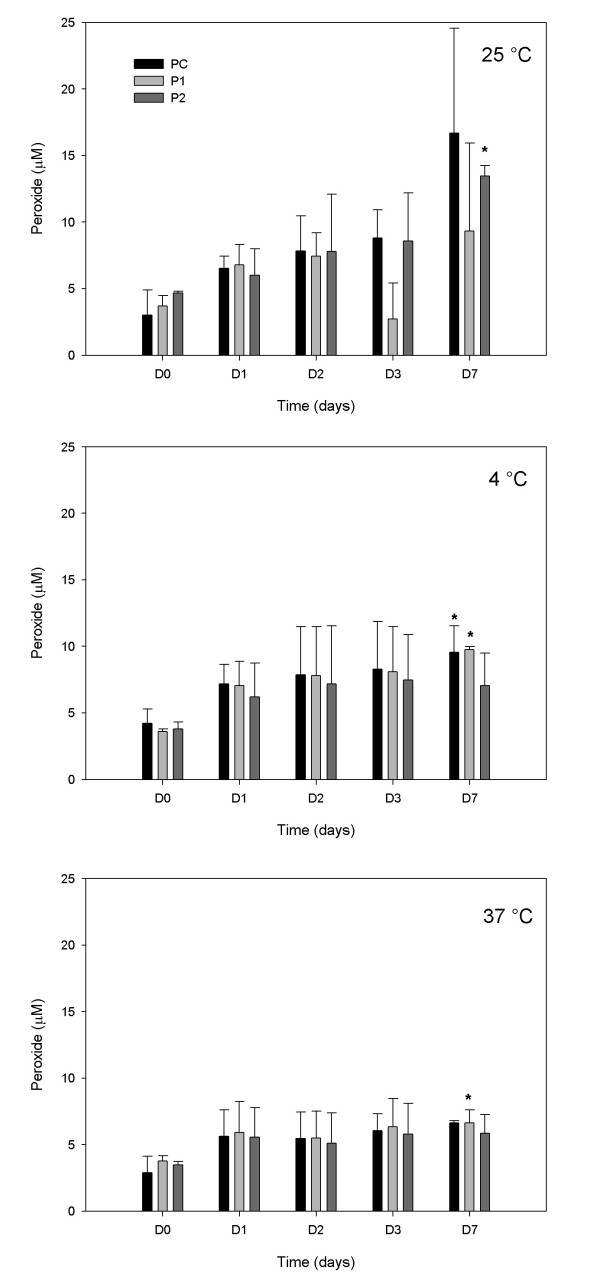
**Peroxide formation in the admixtures**. Bar graphs represent the mean and standard deviation of the peroxide (TBARS) formed in admixtures PC, P1 and P2 stored at 25°C, 4°C and 37°C during seven days of study D0 - D7 (n = 3 lots; *P < 0.05, D7 compared with D0).

PC, P1 and P2 admixtures were tested for peroxide at days D0, D1, D2, D3 and D7. The concentrations of peroxide formed in each admixture over a period of seven days at each temperature are presented in Figure 3. The formulations stored at 4°C and 37°C were kept in total absence of light and stored at 25°C with artificial room light exposure and with photo-protection. Statistical analysis of values obtained at D0 and D7 revealed a significant increase (P < 0.05) in the P1 stored at 25°C (Figure 3A), in PC and P1 at 4°C (Figure 3B) and in P1 at 37°C (Figure 3C). The peroxide values in admixtures with photo protection studied at 25°C on days D0 were: 1.5, 2.5 and 3.7 μM, on day D1: 4.8, 4.8 and 3.5 μM, on day D3: 5.5, 5.10 and 5.3 μM and on day D7: 7.8, 7.7 and 5.2 μM, respectively for PC, P1 and P2 admixtures. The values obtained with photo protection are lower than those observed at the same temperature, under artificial room light exposure (Figure 3A); but increased compared with the admixtures kept at 4°C totally photo protected (cf. Figure 3B).

## Discussion

The TPN admixtures prepared with 2/1 and 4/1 of calcium/phosphorus ratios were free of crystal formation, showing physicochemical compatibility during seven days of storage. Thus, no evidence of incompatibility between calcium and organic phosphorus was observed. It is recommended for preterm an intravenous administration of 200 to 800 mg/kg/day of calcium gluconate and 47 to 70 mg/kg/day of phosphate, concentrations closer to that observed in the uterus. These quantities are difficult to be obtained using the inorganic source due to the low solubility of these ions in aqueous solution [5,25]. In order to maintain the biochemical and hormonal homeostasis and a suitable bone mineralization in preterm infants, the calcium/phosphorus ratio must be greater than 1. The inversion of this ratio can cause an increase in the secretion of parathormone, which leads to loss of phosphate in urine and osteopenia [5].

Clinical research has been showing the advantages in bone mineralization using organic phosphorus [4,18,26-28]. However, in addition to the concern of offering a clinical efficient TPN admixture, the physicochemical the stability of these formulations need to be considered and has been discussed [16,29,30]. Thus, in this work formulations were prepared varying the amount of calcium with fixed amount of phosphate to test the stability of organic phosphorus in the presence of high concentrations of calcium in admixtures containing lipid, vitamins and trace elements. Despite the TPN form a lipid emulsion, which may be subject to a process of physical instability, several metabolic benefits are associated with the administration of TPN admixtures over 2 in 1, such as the prevention of hyperglycemia and the essential fatty acids lack, less manipulation in the infusion line and a lower osmolarity of the final emulsion.

The physical stability of the lipid emulsions can be improved by using surfactants that form a coalescence energy barrier that carries electric charges around the dispersed liquid, aiming at decreasing the surface tension and increasing the repulsion force between the dispersed liquids [31]. In the case of TPN admixtures containing egg lecithin as anionic emulsifier, the lecithin produces a negative charge around the lipid globules through the ionization of the phosphate groups. Any positively charged ion, such as calcium, could cause irreversible instability in this system, neutralizing the negative phospholipids droplets and promoting coalescence [8].

Therefore, the pH of the final admixture is important as a factor that directly influences the ionization of the lipid globule phospholipids, and also interferes in the dissociation of ions in solution. Many factors determine the final pH of the TPN admixture [9], as composition, final concentration of the amino acids, type and final concentration of the phosphate, cysteine addition, peroxidation and final glucose concentration. The pH for pediatric TPN admixtures ranges from 5.0 to 6.0, and the admixtures contain high concentration of amino acids such as HCl cysteine, histidine and arginine, as well as a high concentration of glucose [16,32]. The pH of the admixtures studied remained within this range with no significant alterations over time, showing that the high calcium/phosphorus ratio in the admixture and the temperature of storage had no adverse effect on pH.

Among some of the clinical disorders associated with the parenteral infusion of an unstable lipid emulsion the production of hepatic accumulation of fat associated with oxidative stress, liver injury, and a low-level systemic inflammatory response can be mentioned [33]. In this work it the stability of TPN admixtures with a high concentration of divalent ions was studied. The maximum globule size observed, shown in Table 4, represents a vanishingly small percentage when compared to the total numbers of globules measured, which is the reason because a percentage of this range cannot be visualized in Figure 2. The values observed were well below the recommended limit; however, the increased globules could occur sporadically, that is why it would be important to connect filters in line with the infusion equipment in order to ensure uniformity in the size of the lipid globules infused in these preterm infants. The TPN 1.2-μm filters significantly reduce the total number and concentration of enlarged fat globules and effectively remove rigid crystalline particulates [34]. The other size parameters quantified through OM showed a profile similar to the mean diameter, with no significant alteration during the study. OM is a recognized technique for the qualitative evaluation of TPN admixture stability and its profile, since many randomly selected fields can be considered in the analysis. However, it fails to predict the quantitative evaluation of the lipid stability (PFAT_5_) of the admixtures overall due the small amount of sample employed in the OM analyses. So, these results need to be considered within the limitations of the technique and require confirmation by light obscuration.

Visual inspection is a simple method to evaluate the LE physicochemical compatibility, but extremely important. Color alteration, presence of precipitate and instability phases were evaluated in this step and confirmed microscopically. Color alteration in TPN is well known due to the Maillard reaction and oxidation of some vitamins [6,14]. It is undesirable because it may represent a loss of bioavailability, due loss of some nutrient [5]. In the present study the color alteration that occurred was unrelated to the concentration of calcium, since the PC admixture (without calcium) showed the same change as P2. Color alteration was observed in admixtures stored at 25°C and 37°C, but no alteration was observed when stored at 4°C. Color alteration is related not only to temperature, but also to light, which is the reason for the great difference in color between bags kept at 25°C, in room temperature, and at 4°C, in the refrigerator. In the bag stored at 25°C with photo protection, no color alteration was observed during the days of study (data not shown). Although the infusion of parenteral admixture must be at room temperature, these results indicate that the ideal storage condition is at 4°C, with photo protection, regardless of calcium. The data suggest that the admixtures should not be kept for a long period at 25°C before use, and that equipment with photo protection should be used.

The maintenance of the sterility of the admixtures studied in this paper was fundamental for us to attribute the alterations to possible incompatibility or lipid emulsion instability events due to the high calcium concentration, and not to changes caused by microbiological contamination.

In neonatology, for central access, the osmolarity level must be kept at around 800 mOsm/L, not exceeding twice the regular serum osmolarity [30,35]. High osmolarity arises from high ion concentrations as well as glucose, and the ionic components may also affect the lipid globule stabilization. In this study, the experimental osmolarities were kept within the recommended range for the central access administration of the admixtures studied; and as expected, no significant differences in osmolarities were observed at different temperatures.

The infusion of oxidized lipids and secondary peroxidation products can be extremely cytotoxic, and may cause many disorders, such as hepatic steatosis [36], hyper triglyceridemia [37], increase in vascular resistance in the lung [17,38], lung remodeling [39] and chronic lung diseases of prematurity [40,41]. TPN is a potential source of oxidants and this is particularly dangerous in preterm infants who are vulnerable to oxidative stress [42,43]. The peroxide formation caused by exposing TPN admixtures to phototherapy is well-known in the neonatology field [23,44,45]. This is corroborated by the demonstration that physical protection of the light incidence in TPN admixtures leads to formation of even lower concentrations of peroxides than the usual photo protection [23,45].

The objective of this study was to evaluate the stability of the formulations with high calcium/phosphorus ratio, so experiments were performed varying only the temperature of storage without light exposure. Despite the fact that all peroxide values measured were very low (Figure 3), the admixtures kept photo-protected and totally in absence of light, presented even lower peroxide values, different from those observed in Figure 3A. This observation reinforces the necessity of photo protection for the TPN admixture infusion equipment.

The calcium and phosphorus presence even at high concentrations did not show a statistically significant change in the peroxides value when compared with the control formulation. Low values of peroxide were measured, comparable to values previously demonstrated by other authors when the formulation was totally photo protected [23,45]. The low peroxide value observed could be attributed to many favorable factors, such as the multilayered bag used to store the formulations [42,46]. It has been shown that TPN admixtures in multilayered bag present less oxidation evaluated by hydroperoxide formation [46,47]. This bag has low oxygen permeation and could be collapsed to eliminate, as much as possible, the oxygen in the bag. Moreover, the presence of amino acids [42,48] in formulations containing trace elements, lipids and vitamins [23] decrease the peroxide formation and its clinical complications [39,42]. Lavoie et al [42] showed that during the 6 hours after TPN preparation the balance between generation and consumption of peroxides is positive, and after this time they presume that the balance changed in favor of the consumption, during their transformation into free radicals via the Fenton-like reaction induced by trace elements present in the PN solution [49].

Although it was shown that the vitamin solutions cause more peroxidation [36,41,42], some vitamins have been shown to have antioxidant effects, as vitamin C [23,39,45,50]. The oxidation of aminoacids and vitamin C could prevent the lipid peroxidation turning the peroxidation undetectable. When the amino acid oxidizes the formulation becomes darkened, as observed in admixtures stored at 25°C exposed to artificial light. The relative protective effect of amino acid in the peroxidation in the presence of vitamins and light for 24 hours was already demonstrated [42].

Antioxidant activity of trace elements is demonstrable in some constituents as selenium and copper [51,52]. However, by evaluation of peroxide in the urine in preterm infants, it was observed that the use of trace elements did not affect peroxide production [41]. Even though, Steger et al [53] had shown by in vitro studies the increase in peroxidation in TPN admixtures associated with trace elements, these results may be due to the long period of analysis up to 30 days [53].

## Conclusion

In conclusion, we have demonstrated that, according to the parameters evaluated, the high calcium/phosphorus concentration was compatible. The AIO admixtures studied were physicochemical stable. The three admixtures maintained low peroxide levels. There was no significant pH alteration during the study period. The preliminary results suggest that the admixtures studied did not have abnormal particle or globule counts, and thus, were compatible and stable by OM, but the fact that visual changes were noted in the color and the extent of the lipid film formation in the admixtures stored at 25°C or 37°C may suggest otherwise. As our data are preliminary with regard to particle or globule size analysis in neonatal AIOs, they will require confirmation that by light obscuration to confirm these admixtures are indeed compatible and stable, and thus safe for parenteral administration.

## Abbreviations

TPN: total parenteral nutrition; LE: lipid emulsion; PC: control parenteral nutrition; P1: parenteral nutrition 1; P2: parenteral nutrition 2; TSA/TSB: liquid phase composed of sterile soybean casein broth and a solid phase composed of sterile soybean casein agar; CFUs: colonies forming units; OM: optical microscopy; AIO: all-in-one.

## Competing interests

The authors declare that they have no competing interests.

## Authors' contributions

DRO developed the study design under the supervision of VPS and NMV. BWL and VFV performed the microscopic measurements. LMC contributed to the data interpretation and review of the manuscript. VPS and DRO had primary responsibility for writing the manuscript, but all the others authors read, provided comments on the draft, and approved the final manuscript.
